# Columbia Veterinary College

**Published:** 1882-04

**Authors:** 


### REPORTS OF COMMENCEMENTS, SOCIETIES, ETC

### COLUMBIA VETERINARY COLLEGE.

The annual commencement-exercises of the Columbia Veterinary College and School of Comparative Medicine were held in Chickering Hall, Thursday evening, March 30th. The relatives and acquaintances of the students and the many friends of the College crowded the hall—even standing-room commanding a premium. The stage was beautifully decorated with rare exotics and living plants, and, in many instances, the graduates were overloaded with the many floral offerings of their friends. Soltair's orchestra discoursed most excellent music, and everything betokened healthful prosperity and hearty good-will.

The Rev. Dr. Duffy opened the exercises with an appropriate prayer, after which Alex. Hadden, M.D., President of the Board of Trustees, conferred on the graduates the degree of Doctor of Veterinary Science.

The following is the list of graduates:

Edward S. Breeder, New York City; Edward A McLellan, Bridgeport, Conn.; Edward F. Dowd, New York City; Edward W. Douglass, Brooklyn, N. Y.; George F. Bowers, Brooklyn, N. Y.; Edwin M. Fitzgerald, New York City; Wm. A. Soula, Providence, R. I.; Philip Newman, Brooklyn, N. Y.; James W. Hawk, Newark, N. J.; Flavius Josephus Smith, Austin, Texas; David L. Johnston, Scarsdale, N. Y.; Richard A. Finlay, D.V.S., New York City.

Professor E. S. Bates, Dean of the College, awarded the Junior Certifi-tificate of Honor to the members of the Junior Class, who, on final examination in the studies of the Junior year, had scored an average rank of 70 per cent. He congratulated the class on the very satisfactory exhibit which they had made during the first six months of veterinary instruction, and urged the making of greater effort as they advanced in a more intimate knowledge of comparative medical science.

The Junior Certificate was then awarded to the following:

W. E. A. Cuff, New York; W. H. Jackson, Poughkeepsie; H. C. Slee, New York; H. L. Ramacciotti, Australia; Theo. De Clyne, New Durham, N. J.; H. W. Rowland, Metuchten, N. J.; A. D. Sturges, Wilton, Conn.; W. H. Lowe, Little Falls, N. J.; T. E. Shultze, Brooklyn, N. Y.; Geo. H. Browne, New York City; F. A. K. O'Shea, New York City; R. L. Parkin, Brooklyn, N. Y.

Professor J. H. Gunning, awarded the prizes as follows:

Faculty prize, gold medal, valued at $50—best general examination—to E. A. McLellan.

Honorable mention in connection with this prize was made of George H. Bowers, E. M. Fitzgerald, E. F. Dowd, E. S. Breeder and E. W. Douglass. The contest for this prize was very close, all the contestants taking exceptionally high rank.

The silver medal awarded to the Junior student passing best final examination was awarded to Henry C. Slee, of New York, honorable mention being made of A. D. Sturges, of Connecticut.

The Shoeing prize, a silver medal, was awarded to D. L. Johnston, Scarsdale, N. Y.

The Jurisprudence prize, a case of valuable foot instruments, was awarded to E. S. Breder, of New York.

The Pathological prize to Wm. Soula, Rhode Island, consisting of a veterinary surgeon's visiting chest. An Anatomical prize, consisting of a valuable mouth speculum, was awarded E. A. McLellan.

The Coates prize was awarded to H. C. Slee for the best essay on "Knuckling Over."

A students' quintette club furnished some very excellent vocal music, and the exercises closed with a very able and instructive address by E. M. Hunt, M.D., Secretary New Jersey State Board of Health.

The term which has just closed has been the most satisfactory since the organization of the college. Forty-three new students have matriculated during the term. Over one-half of the graduating class were honor men, or took a rank on final examination of 90 per cent. and upward. The classes are now so large that the accommodations of the lecture room are inadequate to provide for the numbers present, and efforts are being made to erect new and more commodious buildings. We chronicle these facts, as they indicate a growing interest in the subject of comparative medicine, which argues well for its future.

---

